# *Streptomyces* Volatiles Alter Auxin/Cytokinin Signaling, Root Architecture, and Growth Rate in *Arabidopsis thaliana* via Signaling Through the *KISS ME DEADLY* Gene Family

**DOI:** 10.3390/plants15010124

**Published:** 2026-01-01

**Authors:** Bradley R. Dotson, Vasiliki Verschut, Klas Flärdh, Paul G. Becher, Allan G. Rasmusson

**Affiliations:** 1 Molecular Plant Biology Group, Department of Biology, Lund University, 223 62 Lund, Sweden; 2 Department of Plant Protection Biology, Swedish University of Agricultural Sciences, 750 07 Alnarp, Sweden; 3 Microbiology Group, Department of Biology, Lund University, 223 62 Lund, Sweden

**Keywords:** 3-octanone, *Streptomyces coelicolor*, *Streptomyces venezuelae*, *KISS ME DEADLY*, plant–microbe interactions, biostimulation, microbial volatile organic compounds, auxin-cytokinin crosstalk, C8 volatiles

## Abstract

Microbial volatile metabolites can enhance plant growth, yet the mechanisms by which plants perceive and transduce these signals are unknown. Growth of *Arabidopsis thaliana* Col-0 seedlings was found to be stimulated by volatiles from the soil bacterium *Streptomyces coelicolor*. To investigate volatile-responding candidate signaling molecules and genes, cultivation of seedlings in gas-phase contact with *S. coelicolor* genotypes was combined with GC-MS and plant transcriptomics. Components potentially involved were further studied using pure compounds and *A. thaliana* T-DNA mutants. Application of volatiles from *S. coelicolor* enhanced the growth of *A. thaliana* seedlings primarily by stimulating lateral root growth rate and inhibiting primary root extension. Concurrently, a family-wide induction of the Kelch-repeat F-box gene family *KISS ME DEADLY* (*KMD*) was observed. *A. thaliana* genotypes with a loss of function for the *KMD* family or other alterations of auxin/cytokinin signaling homeostasis suppressed the root response to both *S. coelicolor* total volatiles and the common microbial volatile 3-octanone. The results reveal a novel function of *KMD*s in mediating plant growth stimulation in response to volatile stimulation that alters auxin/cytokinin signaling and emphasize rhizospheric microbials as potential indicators of soil status to plants.

## 1. Introduction

Plants have many significant responses related to resource foraging, growth direction, and rate, and resource allocation in response to the environment, which, according to some authors, are considered to constitute decisions [1,2,3]. An example of such a response refers to the allocation of resources between defense- and growth-associated processes during plant development. Production of defensive compounds is especially required due to the sessile nature of plants; however, the metabolic cost depletes energy resources that could otherwise support growth and fecundity [4]. Conversely, a decision to increase growth is necessarily tied to benefits that the plant can perceive in the environment. To illustrate, during split-root growth experiments with pea plants (*Pisum sativum*), the plant’s increased root growth was found to be based on an assessment of available nutrients in the local and extended environment. The pea integrated root growth strategy is thus an important system for biological risk evaluation [5].

Integration of external signals is also involved in the interactions between plants and their microbiomes. Beneficial microbes trigger biostimulation in plants through mechanisms such as nutrient solubilization, hormone modulation, and stress tolerance enhancement [6], and also in biocontrol strategies to protect plants from pathogens and pests [7,8]. The signaling between microbials and plants commences via multiple pathways and molecules that have not been characterized, yet collectively make plants modify growth patterns and resource utilization [9,10,11].

Beneficial microbes have been utilized as biostimulants, promoting plant growth, nutrient uptake, and stress tolerance to increase growth and production [6]. Tissue growth occurs through cell division and expansion, both of which are governed by the plant growth hormones auxin and cytokinin, as well as the crosstalk between their signaling pathways [12]. A wide range of microorganisms have been found to stimulate the growth of various plant species, and such biostimulants are increasingly used in agriculture [10,13,14,15]. Biostimulant microorganisms can promote plants metabolically by, for example, improving nutrient access [13,16] or by microbial production of plant hormones that directly intervene in plant growth regulation [13].

However, recently, indirect interactions via emissions of non-hormonal volatiles from soil microbes have been found to influence plant growth regulation [13,17]. For example, the volatiles 2,3-butanediol and acetoin from the soil bacteria *Bacillus* species enhanced the growth of both *Arabidopsis thaliana* and tomato [18,19]. The effect was linked to auxin signaling, as the volatile-induced transcriptional responses in *A. thaliana* included an abundance of auxin signaling and cell wall remodeling genes, and the growth enhancement could be blocked by auxin transport inhibitors [18]. However, the developmental timing of both the plant and the microbe can influence plant responses. For instance, plant growth was enhanced in response to the volatiles from established cultures of the soil filamentous fungus *Trichoderma atroviride*, though volatiles from younger cultures of the same fungus caused growth inhibition in young plants and enhanced defense responses in older plants [20].

Currently, knowledge on plant perception of microbial volatiles and regulation of growth and/or defense is lacking with regard to the plant signaling components involved. Volatile profiles from cultured soil microbes are highly diverse, and plant growth effects depend on both the microbial strain and the culture conditions [21,22]. To this effect, the previously uncharacterized role of a small family of Kelch repeat F-box proteins known as *KISS-ME-DEADLY* (*KMD*) [23,24,25] is shown to function in the plant perception of volatiles from the common soil bacterium *Streptomyces coelicolor.* The downstream KMD-affected plant gene products that, in turn, influence auxin/cytokinin homeostasis and their involvement in the volatile-promoted tissue growth are further characterized.

## 2. Results

### 2.1. Bacterial Volatiles Enhance Tissue Expansion and Modify Root Architecture in Seedlings

**Figure 1:**
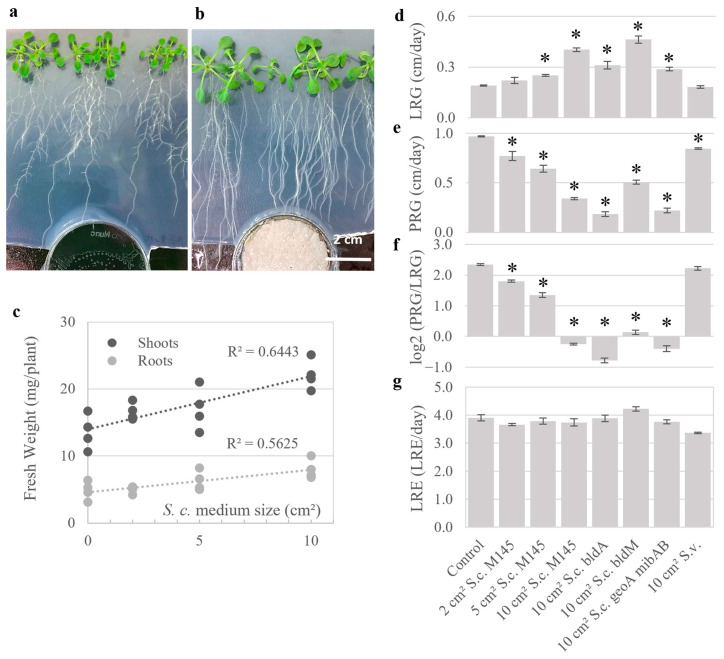
Growth of *A. thaliana* Col-0 in response to *Streptomyces* volatiles. (**a**,**b**) Representative images are shown for seven-day-old *A. thaliana* Col-0 seedlings that have been treated for another six days with gas-phase contact to control plates lacking bacteria (**a**) or to plates carrying *S. coelicolor* M145 grown on 10 cm^2^ of SFM (**b**). (**c**) Fresh weight responses of shoots and roots of seven-day-old Col-0 that have been treated ± volatiles for an additional six days, with linear regression analysis R^2^ values. Dosage of volatiles is expressed as the surface area of bacterial culture. (**d**–**g**) Analysis of root growth responses of seven-day-old Col-0 seedlings treated ± volatiles from different cultures of *S. coelicolor bldA*, *bldM*, *geoA*, *mibAB*, and a *S*. *venezuelae* (*S.v.*) (see Table S1) for two days. (**d**) Lateral Root Growth (LRG) is the average growth rate of the lateral root (cm per day). (**e**) Primary Root Growth (PRG) is the average growth rate of the primary root (cm per day). (**f**) Log2 (PRG/LRG) is the log2 ratio of PRG to LRG. (**g**) Lateral Root Emergence Rate (LRE) is the number of lateral roots emerging from the primary root per day. Bars represent standard error, and asterisks indicate significant differences to the control treatment according to Student’s *t*-test with false discovery rate (FDR) correction for *q* = 0.05.

Seven-day-old *A. thaliana* Col-0 seedlings were exposed for six days to volatiles from cultures of the *S. coelicolor* M145 strain grown on the sporulation agar medium SFM (soya flour and mannitol) (Figure 1a,b). At the highest exposure, treated seedlings showed significant fresh weight increases in both roots (65%) and shoots (63%). The growth effect was linearly correlated to *S. coelicolor* dosage (Figure 1c). Compared to control, volatile-exposed seedlings possessed more expanded leaves, elongated petioles, shorter primary roots, and elongated lateral roots (Figure 1a,b). Quantitative analysis of root growth rate over two days of exposure to *S. coelicolor* volatiles confirmed that the primary and lateral roots responded oppositely to the volatile communication (Figure 1d–f, Table S1). The lateral root growth (LRG) increased to a rate roughly two-fold faster than the control (Figure 1d), while primary root growth (PRG) was strongly repressed compared to the untreated control (Figure 1e). The ratio of primary-to-lateral root growth (PRG/LRG) revealed an apparent shift in resource allocation from primary root growth to lateral root tissue investment in response to the treatment (Figure 1f). However, the rate of lateral root emergence (LRE) was unaffected by the treatment with *Streptomyces* volatiles (Figure 1g). These results indicate that the increased root mass observed can be attributed to a lateral root tissue expansion rather than to an increased number of organs formed.

### 2.2. Seedling Growth Is Modified by Volatiles Independent of Bacterial Developmental Stage

Volatile release changes during bacterial development, and therefore, it was tested whether the volatiles affecting *A. thaliana* seedlings are under developmental regulation in *S. coelicolor* and whether the two major volatiles, geosmin and 2-methylisoborneol, influence the plants. Cultures of *S. coelicolor* mutants of *bldA* (stalled in developmental progression that prevent aerial mycelium formation and sporulation due to lacking an early developmental regulator), *bldM* (stalled in developmental progression to aerial mycelium formation and sporulation due to lacking a later developmental regulator), *geoA mibAB* (lacking the major volatiles geosmin and 2-methylisoborneol), and the control strain of M145 were cultured and used to treat Col-0 seedlings via gas phase. All *S. coelicolor* genotypes induced similar changes in growth rate and root architecture (Figure 1d–g, Table S1, Figure S1a–c,e–g). A minor effect on lateral root growth was observed upon treatment with the *geoA mibAB* line, as compared to M145, but not in the equally geosmin- and 2-methylisoborneol-deficient *bldM* mutant, meaning that these volatiles are not responsible for the change in growth pattern. Likewise, the changes in root architecture occurred without regard to the presence of sucrose in the plant medium (Figure S1h,i), indicating that plant internal metabolic resources were not active determinants of the root architecture shift in our assays.

### 2.3. Screening of Volatiles from Different Isolates

To identify the compound or compounds potentially stimulating *A. thaliana* growth, the correlation between volatile profiles emitted from different *Streptomyces* isolates and their effects on plant seedlings was investigated. The analyzed cultures included *S. coelicolor* isolates M145, *bldA,* and *bldM,* as well as *S. venezuelae*, which, when cultivated on its regular sporulation medium (MYM; maltose, yeast extract, and malt extract), resulted in little or no volatile effect on the growth of *A. thaliana* seedlings (Figure 1d–g and Figure S1d, Table S1). Volatiles were collected from the headspaces of *Streptomyces* cultures for a period of 24 h and analyzed using GC-MS (Table 1). The profiles of volatile compounds from *Streptomyces* cultures were evaluated to identify compounds that may induce root growth responses (Table 1). Small amounts of 3-octanone and two isomers of chalcogran were found in the headspace of growth-inducing *S. coelicolor* cultures grown on SFM, but not in non-inducing *S. venezuelae* that was grown on MYM, suggesting these compounds might contribute to the stimulation of plant growth (Table 1). Despite detecting 3-octanone in various headspace collections from different *S. coelicolor* isolates, 3-octanone could not be detected when M145 was grown on a different batch of SFM or on GA medium. Previously, 3-octanone has been described in two other *Streptomyces* species [26] and its growth-stimulating effect on plants has also been demonstrated when released from other bacteria or the plant-symbiotic fungus *Trichoderma* [27,28,29]. Therefore, this compound was included in further investigations. The compounds acetoin and 2,3-butanediol were specifically targeted as they have previously been found to have growth effects on *A. thaliana* when released from bacteria [30]. However, these compounds were undetectable in all *Streptomyces* headspace samples. Other compounds identified have previously been observed among volatiles from various soil microbial species [21]. 

### 2.4. Plant Hormone Mutants Have Altered Responses to Volatiles

**Figure 2:**
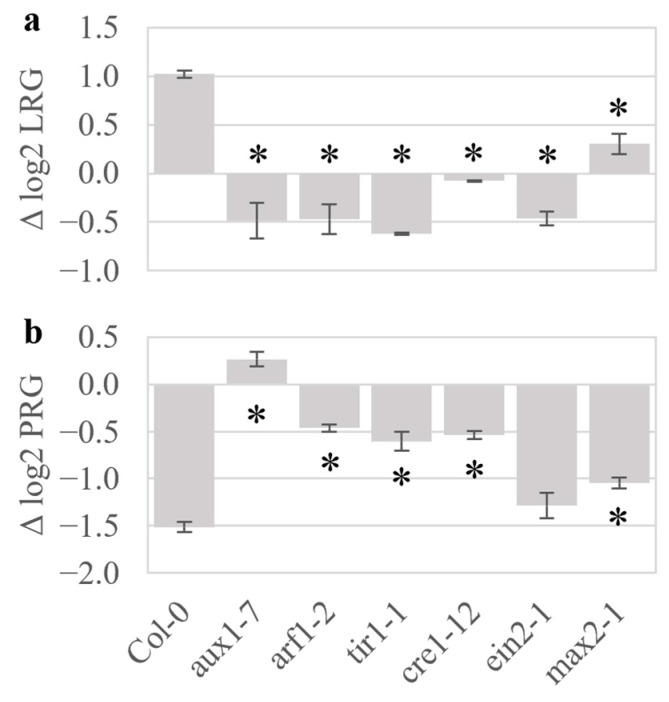
Growth of *A. thaliana* wild-type and hormone mutants in response to *S. coelicolor* volatiles**.** Seven-day-old seedlings of wild-type and hormone mutants were treated for two days with volatiles from *S. coelicolor* M145 grown on 10 cm^2^ of SFM sporulation medium. The figure shows log2 ratios of growth ± volatiles for primary ((**a**); Δ log2 LRG) and lateral ((**b**); Δ log2 PRG) roots. Bars represent standard error, and asterisks indicate significant differences to Col-0 according to Student’s *t*-test with FDR correction for *q* = 0.05.

Mutants for central signaling genes in several hormone pathways of *A. thaliana* were screened to identify signaling processes involved in the plant response to *S. coelicolor* volatiles. For each plant genotype, the log2 root growth rate ratio for the presence and absence of *S. coelicolor* M145 volatiles was determined separately for lateral root growth (Δ log2 LRG) and primary root growth (Δ log2 PRG). This allowed normalization of the responses in the phenotypically diverse sets of mutants (Figure 2). Exposure of Col-0 to the volatiles caused a 2-fold increase in lateral root growth rate, and a 2.8-fold decrease in primary root growth rate during two days of treatment. In contrast, auxin and cytokinin response mutants, including the cytokinin-resistant mutant *cre1-12*, the auxin-response transcription factor *arf1-2*, the auxin influx transporter *aux1-7,* and the F-box receptor for auxin *tir1-1*, were overall unaffected by the volatiles after two days of exposure. However, observation of morphology after six days (Figure S2) indicated that the growth response in *tir1-1* may be merely delayed. Unlike the loss of sensitivity in the cytokinin and auxin mutants, the ethylene signaling mutant *ein2-1* lacked the volatile-induced lateral root growth increase, but the primary root inhibition was retained. Additionally, the strigolactone-insensitive mutant *max2-1* showed less pronounced reductions in both the primary and lateral root growth effects. Mutants for other hormonal pathways, *bri1-4, gai-1*, *and abi1-1* were also tested, but had mild to no discernible reductions in the volatile-induced growth effects (Figure S2). Further verification of auxin involvement was carried out with the auxin response reporter DR5::GFP, which responds in a dose-dependent manner to concentrations of auxin. Analysis of the DR5::GFP root tips showed alterations of auxin distribution in the root tip upon treatment with *S. coelicolor* volatiles (Figure S3). Finally, seedlings of Col-0 were grown on plates containing 3 µM of the cytokinin benzylaldehyde (BA) to heighten cytokinin signaling and were further challenged with volatiles from *S. coelicolor*. This comparison demonstrated that exogenous exposure to BA could suppress the volatile effects on lateral roots (Figure S4). The results thus clearly indicate that the primary and lateral root growth responses to *S. coelicolor* volatiles involve both the auxin and cytokinin response pathways.

### 2.5. The Plant Response to Bacterial Volatiles Shows Similarity to Other Biotic Responses

RNA was extracted from control and *S. coelicolor* volatile-treated seedlings and analyzed by transcriptome sequencing (RNA-Seq). A total of 18,493 transcripts were identified, of which 26 were significantly differentially expressed with a ratio of at least two-fold (Dataset S1). To determine the biological relevance of the response to *S. coelicolor* volatiles, the RNA-Seq expression profile of the 26 significantly responsive genes was compared to the public *A. thaliana* gene expression datasets using the Genevestigator signature search tool (Table 2, see Dataset S2). The highest profile similarity was found to the *A. thaliana* response to volatiles from the rhizobacterium *Serratia plymuthica* HRO-C48 [31]. Other similar datasets were for root colonization by the beneficial *Ascomycota* endophyte *Colletotrichum tofieldiae* and the closely related pathogen *Colletotrichum incanum* during phosphate starvation [32]. The RNA-Seq profile observed here is thus consistent with previous biotic treatments of *A. thaliana.*

### 2.6. Gene Ontology Analysis of RNA-Seq Data Identifies Initial Plant Reprogramming Components

Investigations of gene ontology (GO) were carried out by non-parametric analyses of fold changes for all 18,493 transcripts using the programs Gorilla and Genevestigator (Table 3a). The combination of analyses identified the GO bins of “Negative Regulation of Cytokinin-Activated Signaling Pathways” and “Biological Process” with the lowest FDR *q*-value, followed by “Response to Stimulus”, “Cell Wall Organization”, and “Regulation of Phenylpropanoid Metabolic Process”. Two GO bins, “Negative Regulation of Cytokinin-Activated Signaling Pathways” (Genevestigator) and “Regulation of Phenylpropanoid Metabolic Process” (Gorilla), were both highly enriched in a small family of kelch repeat F-box genes *KISS ME DEADLY* (*KMD1-4*) (Table 3b, Dataset S2). Moreover, the “Cell Wall Organization” GO bin mainly contained pectin degradation and extensin-like proteins, typically involved in cell wall loosening associated with growth changes [33]. Cytokinin- and auxin-associated GO bin responses to *Streptomyces* volatiles were also compared to previously reported responses to exogenous auxin or cytokinin, but no similarities were found (Table S2). The latter result suggests that the effect of the volatiles resides in the response pathways, downstream of the hormones themselves.

### 2.7. Transgenic Plants Deficient in KMD and Associated Cytokinin Response Proteins Are Unresponsive to Volatiles

**Figure 3:**
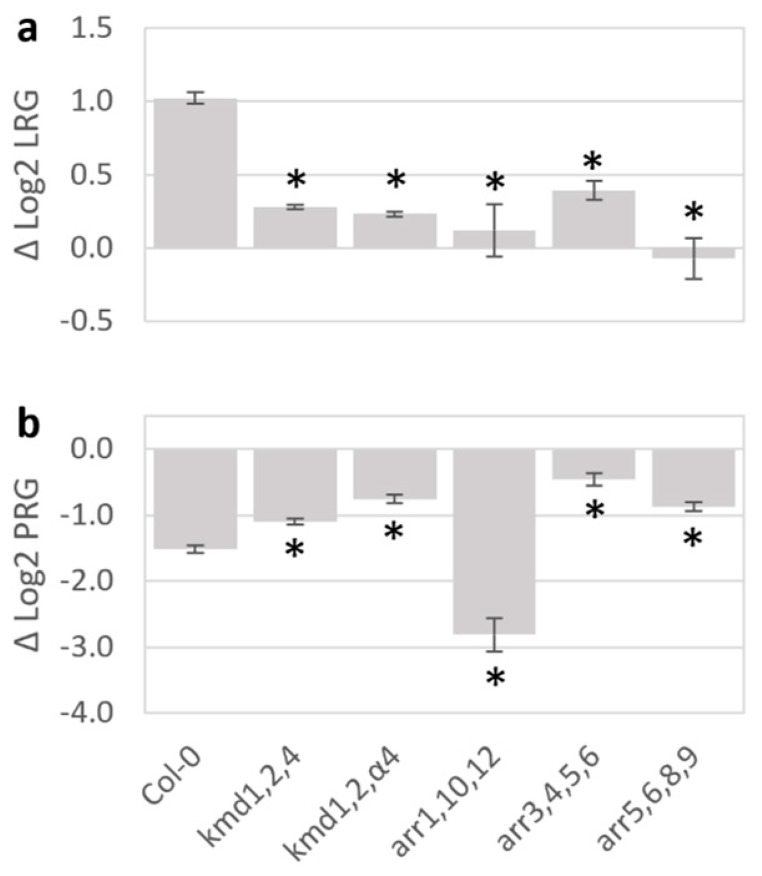
Volatile responses of mutants for *KISS ME DEADLY* and associated hormone response genes. The figure shows log2 ratios of growth ± volatiles for lateral roots ((**a**); Δ log2 LRG) and primary roots ((**b**); Δ log2 PRG) of Col-0, and genotypes deficient and/or suppressed for *KISS ME DEADLY* genes and associated response factors. Seven-day-old seedlings were exposed for two days to volatiles from *S. coelicolor* M145 growing on 10 cm^2^ of SFM. Bars represent standard error, and asterisks indicate significant differences to Col-0 according to Student’s *t*-test with FDR correction for *q* = 0.05.

The KMD protein family consists of four regulatory F-box proteins that target cytokinin regulatory proteins for ubiquitination-mediated degradation, the transcription factors Type B-ARRs (ARR1, 10, and 12), and TCP14 [23,24,25,37]. In addition, the KMD proteins target a set of proteins that function in the initiation of the phenylpropanoid biosynthesis pathway [38]. It was found that the *A. thaliana* triple mutants *kmd1,2,4* and *kmd1,2,α4* had suppressed lateral root growth stimulation and primary root growth inhibition in response to *S. coelicolor* volatiles (Figure 3, Figure S5). Additionally, the cytokinin-insensitive triple mutant of the Type-B-ARR genes *arr1,10,12* displayed enhanced volatile sensitivity in the primary root and lacked the volatile-induced increase in lateral root growth. Further, repressor factors of the cytokinin response known as type-A ARR proteins were tested using quadruple mutants *arr3,4,5,6* and *arr5,6,8,9*. Both type-A ARR lines displayed volatile-resistant phenotypes, the *arr3,4,5,6* being especially resistant to primary root inhibition and *arr5,6,8,9* to lateral root stimulation by *S. coelicolor* volatiles.

### 2.8. The Root Growth Response Elicited by the Bioactive Volatile 3-Octanone Depends on KMD and Auxin/Cytokinin Homeostasis

**Figure 4:**
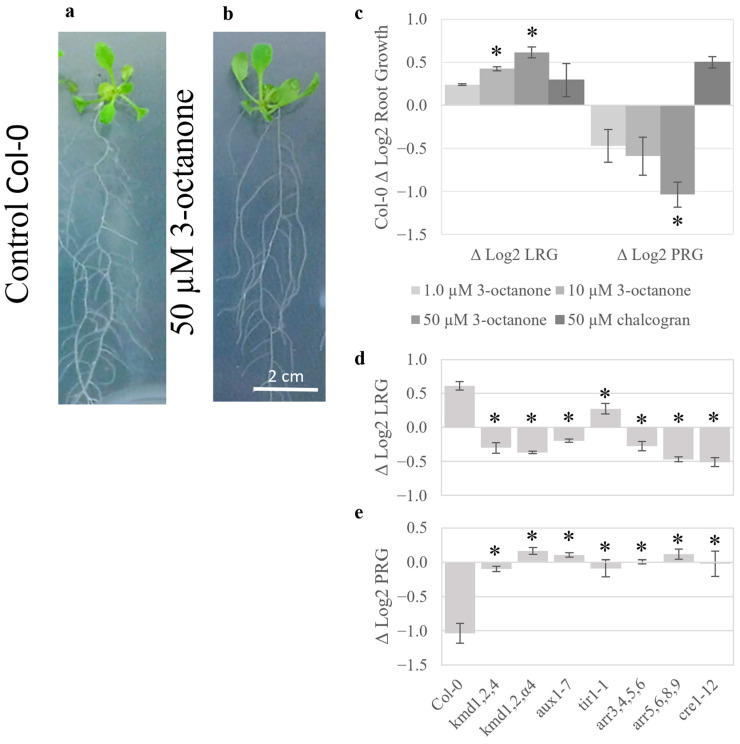
Root growth responses to 3-octanone. Seven-day-old seedlings were transferred to control media or the same media supplemented with different concentrations of 3-octanone or chalcogran. Enhanced growth of roots and shoots can be seen in representative seedlings treated with media containing 3-octanone or solvent control for six days (**a**,**b**). Root growth was quantified during two days of treatment. (**c**) shows log2 growth ratios ± volatile compound (volatile-treated/control) for lateral (Δ log2 LRG) and primary (Δ log2 PRG) roots. (**d**–**e**) show comparisons of Col-0, kmd-genotypes, and hormone response mutants for log2 growth rate ratio ± 50 µM 3-octanone for lateral (**d**) and primary (**e**) roots. Bars represent standard error, and asterisks indicate significant differences for growth ± volatiles (**c**) and to Col-0 (**d**,**e**) according to Student’s *t*-test with FDR correction for *q* = 0.05.

To directly test whether volatile-mediated effects on plant growth involve KMD, 3-octanone, which is known to be bioactive and was present in the *Streptomyces* headspaces that showed plant growth effects, was chosen (Table 1) [27,28,29]. Seven-day-old seedlings were transferred to agar plates containing pure 3-octanone or a solvent control. After six days of treatment, 3-octanone was observed to have induced lateral root growth in Col-0 (Figure 4a,b). Lateral and primary root growth was quantified over two days (Figure 4c–e). Initial studies of Col-0 seedlings with 50 µM chalcogran showed limited effects and were not elaborated on further (Figure 4c). In contrast, Col-0 treated with 3-octanone displayed dose-dependent inhibition of the primary root and stimulation of the lateral root (Figure 4), as observed for total *S. coelicolor* volatiles (Figure 1). Thus, 3-octanone induces a three-fold relative growth rate shift, from primary to lateral roots (Figure 4c). Genotypes deficient in KMD and other signaling components of cytokinin and auxin responses were further tested. All genotypes were resistant to the 3-octanone effect on lateral and primary root growth (Figure 4d,e). The lack of effects, being in stark opposition to the 3-octanone-induced root growth changes in Col-0, indicates that the integral network of *KMD,* as well as cytokinin and auxin homeostasis, must be intact for *S. coelicolor* volatiles or 3-octanone-induced responses to occur.

### 2.9. Integrated Model of the Volatile Response Pathway

**Figure 5:**
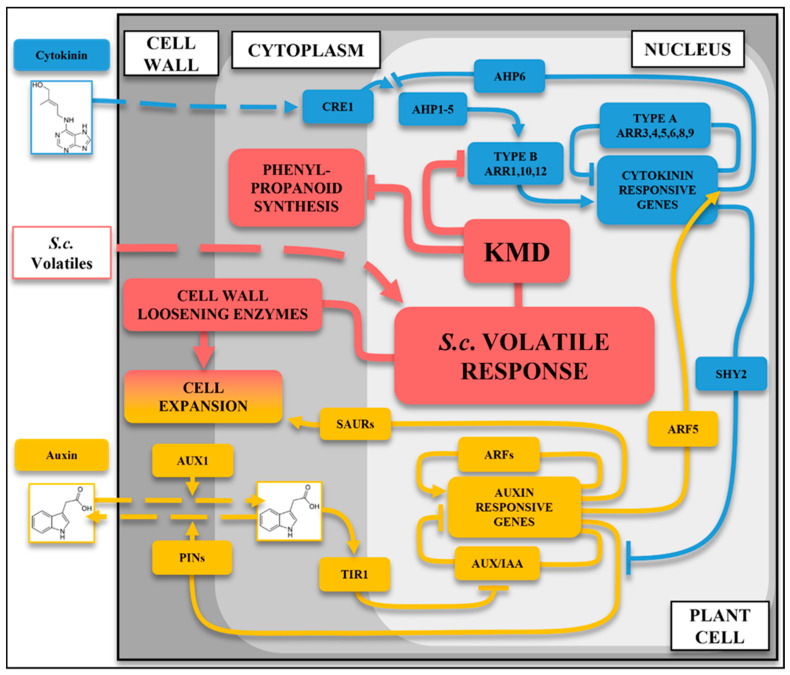
A model for growth induction *by S. coelicolor* volatiles via KMD proteins active at the auxin-cytokinin signaling intersection. Components induced by *S. coelicolor* (*S.c*.) volatiles are colored in red, auxin pathway components in yellow, and cytokinin response pathway components are blue. Connectors ending in arrows or blocks indicate activation and suppression, respectively, and dotted lines represent movement of the compounds to the activation sites. The *A. thaliana* volatile response is caused by *S.c.* volatile perception by the plant cell. This leads to the upregulation of the KISS ME DEADLY (KMD) protein family that suppresses cytokinin signaling through the degradation of Type-B ARR proteins [23], as well as inhibiting phenylpropanoid metabolism [38]. Auxiliary to this, pectinolytic cell-wall-modifying enzymes are enhanced. The reduction in Type-B ARR proteins would lead to decreased expression of *SHY2*, decreasing its suppression of the auxin efflux transporters *PIN*s, leading to increased auxin transport and signaling [12]. Auxin is transported into the plant cell via the influx transporter AUX1/LAX family [12]. Auxin then becomes a ligand of the F-box receptor protein TIR1 that binds and inhibits the AUX/IAAs transcriptional repressors, preventing their interaction with promoter elements in auxin-inducible genes [39]. ARF transcription factors are instead attracted to these promoters and activate the transcription of auxin-responsive genes, such as the SMALL AUXIN UP RNAs (SAURs) that promote cell expansion [12]. Also induced by ARFs are the *AUX*/*IAA* repressing transcription factors, which then act as a close valve for the system [12]. ARF5 has an additive response to promote the cytokinin signaling suppressor AHP6, which inhibits the cytokinin receptor AHK/CRE1 phospho-transfer to AHP1-5 proteins [12]. The reduced phosphorylation of AHP1-5 leads to inactivation of Type-B ARR proteins and enhanced suppression by ambient Type-A ARRs proteins [12].

Integration of growth phenotyping, transcriptomic analysis, and *Arabidopsis* mutant responses reveals a coordinated response network in which *Streptomyces* volatiles induce *KMD* expression that, in turn, suppresses cytokinin signaling via degradation of type-B ARR regulators [23,25] and phenylpropanoid components [38]. This activity then relieves SHY2-mediated repression of PIN expression and enables enhanced AUX/LAX and TIR1-dependent auxin signaling alongside cell-wall-loosening processes [12,33,39]. This hormonal rebalancing informs the root growth patterning of enhanced lateral root expansion together with constrained primary root elongation, as summarized in Figure 5.

## 3. Discussion

*A. thaliana* is adapted to an extensive range of nutrient availability and has consequently evolved decision mechanisms to adapt to variable local soil environments [40]. Root growth investments in soil niches with high microbial activity have previously been shown to improve plant overall growth by enhancing plant nutrient status, pathogen resistance, and mitigating stress responses [41,42]. It is therefore expected that higher levels of microbial activity would be cues for plant decisions to locally enhance root tissue investments [43].

The 3-octanone volatile organic compound has previously been shown to be one of many metabolites released from in vitro *Streptomyces* cultures [26], but has also been isolated from other rhizospheric microorganisms such as *Proteobacteria*, *Ascomycota*, and *Basidiomycota* [28,44,45,46]. However, 3-octanone has not been shown to be produced by vascular plants. Along with 1-octen-3-ol, 3-octanone belongs to a class of eight-carbon (C8) compounds that possess bioactivity in organisms like bacteria, fungi, and plants. Notably, 3-octanone is a conidia-inducing signal in the fungal genus *Trichoderma* [47], which includes several plant-symbiotic species [48]. *Trichoderma*-produced 3-octanone has been shown to promote the growth of plants such as *A. thaliana* and willow [27,28,29]. In contrast, 1-octen-3-ol induces defense responses in *A. thaliana*, suppresses growth and development of fungi, and reduces growth, chlorophyll accumulation, and seed germination in *A. thaliana* [49,50]. The bioactive C8 compounds share structural similarities with the defense regulators C9 oxylipins, and both are derived from linoleic acid [51]. Unlike the C9 oxylipins, there are currently no known components mediating the perception or response to C8 compounds in higher plants.

The bioactive volatile signal 3-octanone is consistently present in the headspace of *S. coelicolor* cultures grown on SFM sporulation media. This happens regardless of bacterial development because volatiles from *S. coelicolor* mutants stalled at different developmental states still include 3-octanone and influence the *A. thaliana* root growth. Based on the presence of 3-octanone in the *S. coelicolor* volatile headspace and the similarity in effects of 3-octanone and *S. coelicolor* volatiles on *Arabidopsis* root growth, we conclude that the action of 3-octanone is sufficient to explain the volatile elicitation effect on the root growth, although we cannot rule out that other volatiles in the *Streptomyces* headspace had additional effects. These data thus present the first evidence linking C8 volatiles to *KMD* expression and root growth modulation.

Volatiles from *S. coelicolor* cultured on the *S. venezuelae* sporulation medium (MYM) had little effect on *A. thaliana* root growth, whereas *S. venezuelae* enhanced lateral root growth only when cultured on *S. coelicolor* sporulation medium (SFM) (Figure S1). This indicates that the *Streptomyces* medium is a critical factor for volatile-mediated plant growth promotion. Likewise, in vitro studies of several other rhizospheric bacteria have revealed that plant growth promotion by volatiles depends on the culture media used for the microbe [21]. Both 3-octanone and chalcogran have been identified as signature volatiles from fungal cultures on media high in linoleic acid [52,53]. This fatty acid is highly abundant in soya flour, which is a main component of the SFM (~1.4 g linoleic acid per L) [54,55]. Unsaturated fatty acids, including linoleic, tend to be degraded rapidly in soil by abiotic conditions and by microbes [56]. To plants, soils that are enriched in linoleic acid-associated microbial volatiles may indicate a richness in organic compounds and availability of new nutritional resources for plant roots to harvest.

To investigate factors regulating the *A. thaliana* response to volatiles from *S. coelicolor*, exposed seedlings were analyzed by RNA-Seq. Transcriptional analysis revealed a differential expression pattern that displayed similarities to previously reported *A. thaliana* responses to beneficial microorganisms *Serratia plymuthica* HRO-C and *Colletotrichum tofieldiae* [31,32]. Two GO approaches specifically indicated the gene family *KISS-ME-DEADLY* (*KMD*) as contributors to the *S. coelicolor* volatile-induced gene response profile in *A. thaliana*. The GO bin with the lowest FDR *q*-value, “negative regulation of cytokinin-activated signaling pathways”, contains three out of the four genes in the *KMD* family. This associates the volatile response with KMD-mediated suppression of type-B ARR cytokinin signaling factors [23]. Additionally, the entire family of *KMD* genes is included in the likewise responsive bin “Regulation of phenylpropanoid metabolic process”, which denotes the association of *KMD* genes with suppression of the phenylpropanoid biosynthesis pathway, potentially lowering the capacity for defense, such as pathogen responses or herbivory deterrence [38].

Concurring with transcript analysis, investigations into broad hormone regulation by *S. coelicolor* volatiles response were analyzed by genetic perturbations in the plant hormone pathways. Mutant lines impaired in auxin response (*aux1-7*, *arf1-2*, and *tir1-1*) and cytokinin (*cre1-12*) signaling had the smallest initial root growth response to *S. coelicolor* volatiles. Consistent with the GO analysis, mutations to KMD family function suppressed both primary and lateral root responses to *S. coelicolor* volatiles as well as to the pure 3-octanone. The mutant lacking the targets of KMD function, Type B ARRs (ARR1,10,12), also lacked lateral root growth enhancement but retained a strong primary root inhibition, possibly due to volatile-induced KMD proteins suppressing remaining Type B ARRs, such as ARR20 [23]. Mutations in the cytokinin response-suppressive factors Type A ARRs (*arr3,4,5,6* and *arr5,6,8,9*) also suppressed the *S. coelicolor* volatile growth response, further emphasizing that repression of cytokinin signaling is involved in mediating the *S. coelicolor* volatile-induced plant growth decision. Previous research has characterized the downstream signaling effects of KMD, which causes a shift in the cytokinin-to-auxin signaling toward more auxin-like responses (e.g., increased cell expansion and enhanced lateral root growth) and promotes synthesis of defense-associated phenylpropanoids [23,25,57]. This study identifies an upstream cue for the *KMD* family, which therefore can be placed as an upstream regulator of auxin/cytokinin crosstalk for effects on root growth plasticity in response to a biotic interaction. A model is suggested for the consecutive steps from KMD activation, including changes in relative tissue sensitivity to auxin and cytokinin that promote wall loosening and cell expansion [12,33]. The model is consistent with known crosstalk events that balance the auxin/cytokinin homeostasis. One such example involves *SHORT HYPOCOTYL2* (*SHY2*), which is induced by positive regulators of cytokinin signaling of the Type-B *ARR* family [12,23,25]. *SHY2* suppresses PIN-mediated auxin membrane transport, causing a local auxin accumulation in cells with high amounts of cytokinin-induced signal components [12]. When auxin signaling is activated, degradation of SHY2 via the SCF^TIR1^ ubiquitination pathway and promotion of *AHP6* by ARF5 decrease the cytokinin suppression of auxin transport [12], thus mediating a developmentally tight control of growth. KMD family proteins induce degradation of Type-B ARR 1,10,20 proteins [23,25], so inactivation of the *KMD gene*s leads to accumulation of Type-B ARRs and promotes the cytokinin response [23]. A volatile-induced KMD-mediated loss of cytokinin signaling would decrease the suppression of auxin signaling (e.g., via the *ARR1,10*-dependent auxin-suppressing gene *SHY2*) and enhance *PIN* expression and tissue elongation [12]. Supporting such a scenario for the root growth effect of *S. coelicolor* volatiles, the GO analysis also indicated induction of genes for cell wall loosening enzymes, which are associated with cell expansion [33]. The lack of transcriptional differences between control and volatile-treated plants for auxin-responsive genes in signaling, metabolism, and transport further indicates that the observed promotion of lateral roots is a result of *S. coelicolor* volatiles decreasing cytokinin-induced suppression of auxin signaling.

## 4. Conclusions

The general release of the identified active volatile 3-octanone from multiple species of bacteria and fungi, and its dependence on growth substrate composition [58,59] makes this volatile a prime candidate signal for mediating soil information to the plant, relatively independent of the specific microbial composition, yet possibly reflecting the quantitative microbial soil status. The 3-octanone is demonstrated to have a profound effect on the *A. thaliana seedlings* to promote lateral root growth, and consequently, total growth. The growth stimulation depends on the activation of KMD genes that lead to a modified sensitivity to the growth hormone cytokinin. This leaves the potential utilization of 3-octanone for the biostimulation of agricultural plants as an intriguing possibility; however, more information about the downstream effects of 3-octanone application must be investigated beyond the early plant growth stage.

## 5. Methods

Details about the statistical analysis data are described in Dataset S3. Genetic stocks used are described in Table S3.

### 5.1. Plant Growth System

Seeds of *A. thaliana* were surface-sterilized in microtubes with 70% ethanol, then 50% bleach, and finally washed three times with diH_2_O and stratified for 2 days at 4 °C [60]. The seeds were then placed on vertical 12 × 12 cm 0.8% agar (*w*/*v*) plates containing ½ Murashige and Skoog (MS) [61] basal medium (pH 5.50–5.65) supplemented with 88 mM sucrose, unless otherwise stated. Agar plates supplemented with compounds (such as 3-octanone) were prepared similarly, except that the compounds were added from sterile filtered stocks in dimethyl sulfoxide (DMSO) to the autoclaved growth media after allowing the media to cool to <60 °C. Thus, the volatile-supplemented agar plates also contained 0.01 µM DMSO, which was also used in control plates. Prepared agar plates were used immediately. The plates with seeds were incubated in a growth chamber (16 h light period, 120 µE m^−2^ s^−1^, 25 °C).

Vertical plates with growing plants were modified by removing the bottom 4 cm of the agar medium and placing a 3.5 cm Petri dish inside. Variably sized disks of sporulating *Streptomyces* cultures were placed onto the 3.5 cm mini-Petri dish after 7 days of plant growth. The plant-bacterial plates were then scanned with a Ricoh Aficio MP C5000 scanner every 2 days over a 6-day period to measure growth differences. After six days of exposure, the plates were photographed using an iSight camera (Apple). All image processing was performed by ImageJ 1.48v (National Institute of Health) [62].

### 5.2. Bacterial Growth

Approximately 5 µL of a spore suspension was spread over sporulation media, which was soya flour–mannitol media (SFM) for all isolates of *S. coelicolor*, starch and KO3 minimal media (GA), and maltose–yeast extract–malt extract (MYM) media supplemented with 0.2% trace minerals for *S. venezuelae* [54,63,64]. Soya flour used for SFM media was sourced from Risenta brand soya flour, Stockholm, Sweden. *Streptomyces* cultures were allowed to grow to a dense lawn of mycelium over a period of 8 days at 30 °C before being used for volatile treatments.

### 5.3. Analysis of Tissue Growth

Primary and lateral root growth was quantified over a period of two days by ImageJ analyses of scanned photos. Lateral root growth was assessed using the largest three existing lateral roots per seedling, and primary root growth was assessed per seedling. Experimental growth rates were compared internally to the overall lateral root growth/primary root growth (log2 LRG/PRG). The number of emerging lateral roots per day (LRE) was calculated per primary root present. Alternatively, volatile treatments were compared to control settings for lateral root growth (Δ log2 of volatile-treated LRG to log2 control LRG) and for primary root growth (Δ log2 of volatile-treated PRG to log2 control PRG). For each measurement of growth, averages from technical replicates of 3 or more biological replicates were pooled into 3 biological replicates and used to compare between experimental and control. The significance of growth differences was tested by Student’s *t*-test and verified with an FDR *q*-value of 0.05 [65].

### 5.4. Volatile Collection and Analyses

Volatiles were collected from 8-day-old *Streptomyces* cultures over a period of 24 h by adsorption to air filters made of Teflon tubes (4 × 40 mm), holding 50 mg Porapak Q (80/100 mesh; Alltech, Nicholasville, KY, USA). Adsorbed volatiles were desorbed by eluting from the filters with 500 µL re-distilled n-hexane. Two µL of collected volatiles were resolved by gas chromatography-mass spectrometry (GC-MS; 6890 GC and 5975 MS; Agilent Technologies Inc., Santa Clara, CA, USA) using a polar DB-WAX capillary column (30 m × 0.25 mm × 0.25 μm; J&W Scientific, Folsom, CA, USA) using helium as carrier gas at a flow rate of 35 cm/s. The oven temperature was held at 30 °C for 3 min, increased with a ramp rate of 8 °C/min to 225 °C, and held for 10 min. Compounds were then subjected to mass spectrometry in a GC-MS (6890 GC and 5975 MS; Agilent Technologies Inc., Santa Clara, CA, USA). Compound identities were determined according to mass spectra libraries at SLU Alnarp and the library of the National Institute of Standards and Technology (NIST; www.nist.gov), retention times (using the Kovats’ index [66] NIST database for polar columns) and by co-injection of authentic compound standards (Sigma, Hong Kong, China).

### 5.5. RNA Extraction

Single replicate experiments with volatile and control treatments were independently repeated three times using populations of approximately 20 seedlings. Seedlings were exposed to control or *S. coelicolor* M145 volatile conditions for 2 h and flash-frozen in liquid N_2_. Approximately 100 mg of tissue was placed into a microtube and then ground with plastic pestles to obtain a homogenized sample. RNA was extracted according to the protocol of the RNeasy Kit (Qiagen, Venlo, The Netherlands) and treated with the DNA-free DNA Removal Kit (ThermoFisher, Waltham, MA, USA). About 1 µg of RNA from each of the six samples (three control and three volatile-treated biological replicates) was sent to Beijing Genomic Institute (Hong Kong, China) for RNA-Seq analysis using the Hiseq 2000 Illumina sequencing platform and single-end 50 base pair sequencing.

### 5.6. Analysis of RNA-Seq Data

Raw reads were mapped to the genome of *A. thaliana* (TAIR 10) [67] as described [68]. Reads were summarized per gene (including splice variants) and divided by mRNA length to calculate reads per kb million (RPKM), which were log2-transformed. Transcripts identified as non-coding or organellar, as well as lowly expressed genes (less than 15 mapped reads per gene), were removed and excluded from the analyses. Transcripts were then mean expression-normalized according to Willems et al. 2008 [69] for calculation of log2 expression ratios and statistics. Differential expression was tested by Student’s *t*-test and corrected for multiple testing using a false discovery rate (FDR) *q*-value of 0.05 [61]. A list of significantly differentially expressed genes (log2 ratio > 1.0; FDR *q*-value = 0.05) was subject to “Signature Search” using Genevestigator (https://genevestigator.com/gv/, accessed on 1 February 2016) [34]. Additional analyses were performed on the entire list of expressed genes using non-parametric GO analysis using the Gorilla website (http://cbl-gorilla.cs.technion.ac.il/, accessed on 1 February 2016) [35] and the program Genevestigator with default settings (for summary see Dataset S4).

### 5.7. qRT-PCR Confirmation

Four independent RNA samples were analyzed in triplicate using the MAXIMA First Strand Synthesis Kit (K1671; Thermo Scientific, Waltham, MA, USA) according to the manufacturer’s guidelines. The resulting cDNA was quantified by qPCR using the MAXIMA SYBR green kit with 40 cycles in a Rotor-gene Q thermocycler (Qiagen Venlo, The Netherlands), following the settings: 95 °C, for 2 min; and cycles of (95 °C, 20 s; 60 °C, 15 s; 72 °C, 15 s; 81 °C, 20 s); 1 cycle of 50 °C, 1 min. Signals were acquired at 72 °C and 81 °C for analyzing consistency in accumulation, and comparative quantification was performed on data acquired at 81 °C. Three technical replicates were run for each biological replicate. Gene products of the housekeeping gene *UPL7* (At3g53090) and three experimental genes *NIT1* (At3g44310), *PP2AA3* (At1g13320), and *EXP5* (At3g29030) were analyzed (Figure S6). Each reaction had a volume of 20 µL and contained cDNA corresponding to 0.16 ng of DNase-treated RNA. Product identity and specificity were confirmed by melt curve analysis, as well as gel electrophoresis of representative and ambiguous samples. Individual reactions that showed unspecific products were deleted from the analysis. Primers were designed using the program Primer3, and are listed in Table S4 [70].

## Figures and Tables

**Table 1:** Microbial volatile analysis.

	*S.c.* M145	*S.c. bldA*	*S.c. bldM*	*S.v.*	K.I. Exp.	K.I. Pub.	CAS Number	ID Method
**A-unknown**	0.03 ± 0.01	N.D.	N.D.	N.D.	1134	-	-	MS
**B-unknown**	N.D.	N.D.	N.D.	6.8 ± 0.3	1147	-	-	MS
**3-Octanone**	0.15 ± 0.02	2.9 ± 0.8	0.8 ± 0.1	N.D.	1260	1266	106-68-3	MS, KI, RC
**Styrene**	0.26 ± 0.04	2.0 ± 0.4	1.6 ± 0.2	0.3 ± 0.1	1263	1259	100-42-5	MS, KI
**Chalcogran (i)**	0.3 ± 0.1	0.7 ± 0.1	1.0 ± 0.3	N.D.	1357	1343	35401-84-2	MS, KI, RC
**Chalcogran (ii)**	0.4 ± 0.2	1.1 ± 0.1	1.3 ± 0.4	N.D.	1361	1348	35401-84-2	MS, KI, RC
**C§-unknown**	0.36 ± 0.04	N.D.	1.3 ± 0.3	N.D.	1436	-	-	-
**2-Methyl-Isoborneol**	6.4 ± 0.8	3.2 ± 0.3	N.D.	60.7 ± 4.6	1595	1602	2371-42-8	MS, KI, RC
**E-unknown**	0.33 ± 0.04	N.D.	2.7 ± 0.4	N.D.	1610	-	-	MS
**F-unknown**	N.D.	0.7 ± 0.4	N.D.	1.7 ± 1.6	1714	-	-	MS
**Germacrene D**	9.9 ± 1.9	0.9 ± 0.4	N.D.	1.2 ± 0.1	1724	1724	483-76-1	MS, KI
**G-unknown**	N.D.	42.8 ± 3.1	30.8 ± 2.4	N.D.	1732	-	-	MS
**H-unknown**	N.D.	N.D.	N.D.	1.0 ± 0.7	1747	-	-	MS
**Geosmin**	49.1 ± 3.2	6.9 ± 0.2	N.D.	6.2 ± 2.0	1845	1858	19700-21-1	MS, KI, RC
**I-unknown**	1.7 ± 0.5	N.D.	N.D.	4.3 ± 0.4	2150	-	-	MS

Volatile headspace from four different *Streptomyces* genotypes grown on sporulation media was analyzed by GC-MS using a DB-wax column. Volatile compound name, percent of total volatile headspace ± standard error, Kovats’ index, CAS number, and method of identification are listed from each of the *Streptomyces* headspace analyses. The compounds that were identified specifically in the headspaces of growth-inducing cultures are highlighted. Identification (ID) methods are by mass spectrometry (MS), Kovats’ retention index (KI), and authentic reference compounds (RC). Unidentified compounds are listed by letters, and mixed volatile profile peaks of two or more compounds are denoted by §. Data for compound quantities are average percentages of total volatile content ± standard error or not detectable (N.D.).

**Table 2:** Similarity analysis of volatile-induced.

Species	Experiment Description	Experiment Number	Searched Dataset Collection	GEO Number	Similarity Score	Ref.
***Serratia*** ***plymuthica*** **HRO-C**	48 volatile-treated seedlings/mock-treated seedling (24 h after treatment)	AT-00496	ATH1 Microarray	GSE35325	2.6	[31]
***Colletotrichum tofieldiae***	inoculated seedling roots (low P)/untreated seedling roots (sufficient P) (10 days after inoculation)	AT-00754	RNA_SEQ_	GSE70094	2.6	[32]
***Colletotrichum*** ***incanum***	inoculated seedling roots (low P)/untreated seedling roots (sufficient P) (10 days after inoculation)	AT-00754	RNA_SEQ_	GSE70094	2.3	[32]

*Arabidopsis thaliana* RNA profiles. Seven-day-old *A. thaliana* Col-0 seedlings were exposed to a 10 cm^2^ *Streptomyces* culture for 2 h and analyzed by RNA-Seq. RNA-Seq data profiles for genes that were found to be significantly differentially expressed, compared to control (see Dataset S2), and showing a response of at least two-fold, were analyzed by Genevestigator Signature Search against responses in publicly available microarray and RNA-Seq expression datasets using Pearson correlation.

**Table 3:** Gene Ontology analysis.

**(a)** **Program**	**Description of Bin**	**GO Bin Hits**	**GO Bin Size**	**GO Term**	**FDR** ***q*** **-Value**
Genevestigator	Negative Regulation of Cytokinin-Activated Signaling Pathways	3	3	80,037	0.008
Genevestigator	Biological Process	116	22,565	8150	0.008
Genevestigator	Response to Stimulus	52	6661	50,896	0.014
Gorilla	Cell Wall Organization	20	109	71,555	0.071
Gorilla	Regulation of Phenylpropanoid Metabolic Process	4	5	2,000,762	0.09
**(b)** **Gene Number**	**Gene Description**	**Average FPKM Control**	**Average FPKM *S.c.* Volatiles**	**Fold Change *S.c.* Volatiles/Control**	**GO Bin**
At1g80440	Galactose oxidase/kelch repeat superfamily protein; *KISS ME DEADLY 1*	95.8	182.5	1.9	GO:80037; GO:0008150; GO:0050896; GO:2000762
At1g15670	Galactose oxidase/kelch repeat superfamily protein; *KISS ME DEADLY 2*	104.1	171.2	1.7	GO:80037; GO:0008150; GO:0050896; GO:2000762
At2g44130	Galactose oxidase/kelch repeat superfamily protein; *KISS ME DEADLY 3*	67.7	113.2	1.7	GO:0008150; GO:0050896; GO:2000762
At3g59940	Galactose oxidase/kelch repeat superfamily protein; *KISS ME DEADLY 4*	221.4	302.6	1.4	GO:80037; GO:0008150; GO:0050896; GO:2000762

(a) Gene ontology analysis was performed on the total list of 18,493 genes, sorted by fold changes. Resulting GO terms, GO bin hits, GO bin sizes, and descriptions of FDR *q*-values are as provided by Gorilla and Genevestigator, and descriptions are as annotated by AmiGO [34,35,36]. (b) Description of the *KISS ME DEADLY* family of genes identified by GO analyses as a significantly affected group of genes in bins of GO:0080037 and GO:2000762. Fragments per kilobase of exon model per million reads mapped (FPKM), and the complete information can be found in Dataset S1.

## Data Availability

The RNA-Seq data in this study have been deposited in the NCBI GEO under the accession number PRJNA573628. Figures, datasets, and Supplemental Information associated with raw data that supported the findings of this study are available from the corresponding author upon reasonable request.

## Supplementary Materials

The following supporting information can be downloaded at: https://www.mdpi.com/article/10.3390/plants15010124/s1, Figure S1: Growth of *Arabidopsis thaliana* Col-0 in response to *Streptomyces volatiles*; Figure S2: Growth of *Arabidopsis thaliana* hormone mutants in response to *Streptomyces coelicolor* M145 volatiles; Figure S3: *Arabidopsis thaliana* DR5::GFP response to *Streptomyces coelicolor* volatiles; Figure S4: Root growth response of *Arabidopsis thaliana* Col-0 seedlings exposed to *Streptomyces coelicolor* volatiles with cytokinin; Figure S5: Growth of *Arabidopsis thaliana* mutants for *KMD*-associated genes in response to *Streptomyces coelicolor* volatiles; Figure S6: qRT-PCR verification of RNA-Seq expression; Table S1: Growth data for Col-0 response to gas-phase connection to different amounts of each Streptomyces strain; Table S2: Transcriptional comparison of *Arabidopsis thaliana* Col-0 treated with *Streptomyces coelicolor* volatiles to exogenous hormone application; Table S3: Genetic stocks [71,72,73,74,75,76,77,78,79,80,81,82,83,84,85,86]; Table S4: Primer sequences; Dataset S1: RNAseq dataset; Dataset S2: RNA-Seq Differentially Expressed Gene set; Dataset S3: Statistical summary; Dataset S4: Gene ontology analysis summary.
