# Supplementary material for: Streptomyces Volatiles Alter Auxin/Cytokinin Signaling, Root Architecture, and Growth Rate in Arabidopsis thaliana via Signaling Through the KISS ME DEADLY Gene Family
Source: Plants (Basel). 2026 Jan 1;15(1):124. doi: 10.3390/plants15010124 (PMC12788082; doi:10.3390/plants15010124)
Supplement: Supplementary file 1 [file plants-15-00124-s001.zip › Dotson et al 2026 Supplemental.pdf]

Supplemental Data

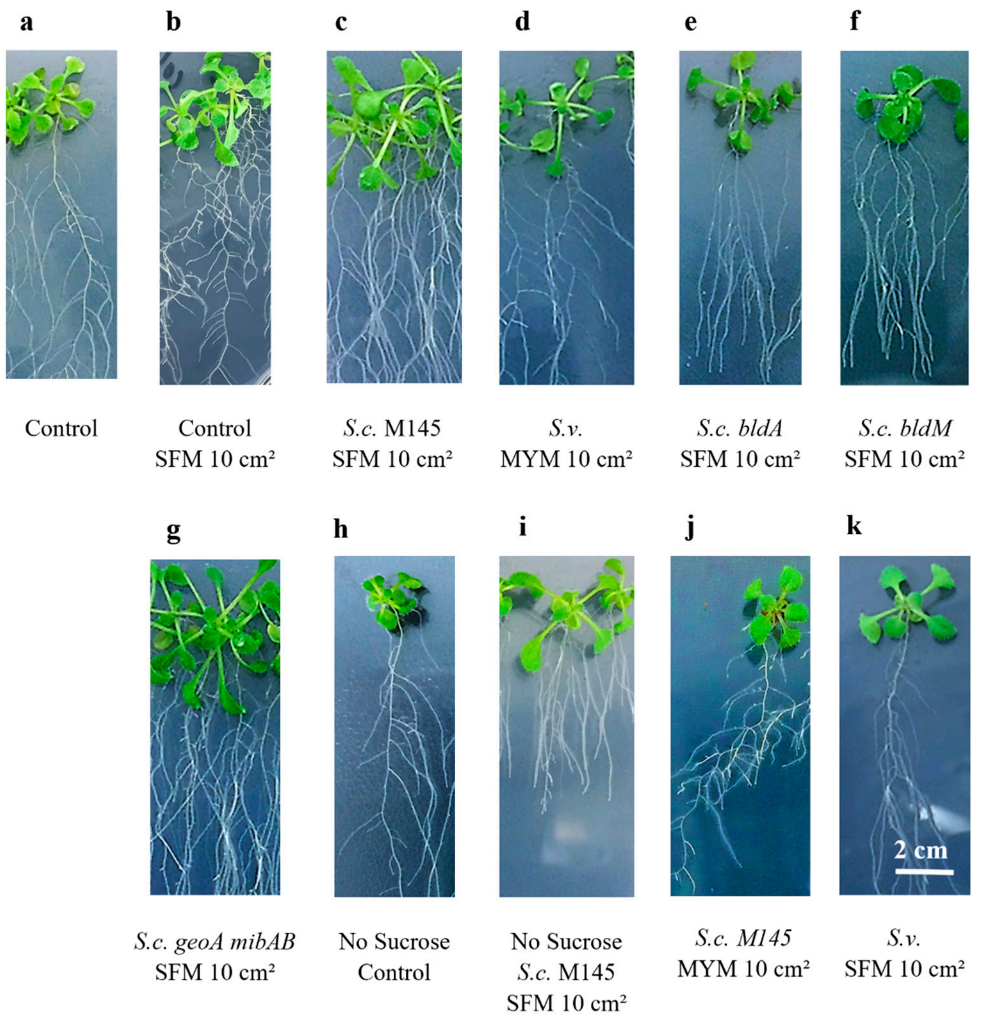

Figure S1. Growth of *Arabidopsis thaliana* Col-0 in response to *Streptomyces* volatiles. Seven-day-old seedlings of *A. thaliana* Col-0 were treated ± gas-phase contact with 10 cm^2 *Streptomyces* agar cultures for six additional days. **a**, Control growth. **b,** control growth with SFM sporulation media alone, **c**, *S. coelicolor* (*S.c.*) M145 wild type on SFM medium. **d**, *S. venezuelae* (*S.v.*) grown on MYM medium. **e-g** Developmental *S. coelicolor* mutants *bldA* (**e**), *bldM* (**f**), and *geoA mibAB* (**g**) cultured on SFM. **h-i**, seedlings grown on plant media without sucrose and treated ± volatiles from *S. coelicolor* M145 cultured on SFM. **j-k**, Seedlings grown as in **a-g** and treated with volatiles from M145 cultured on MYM (**j**) and *S. venezuelae* cultured on SFM (**k**).

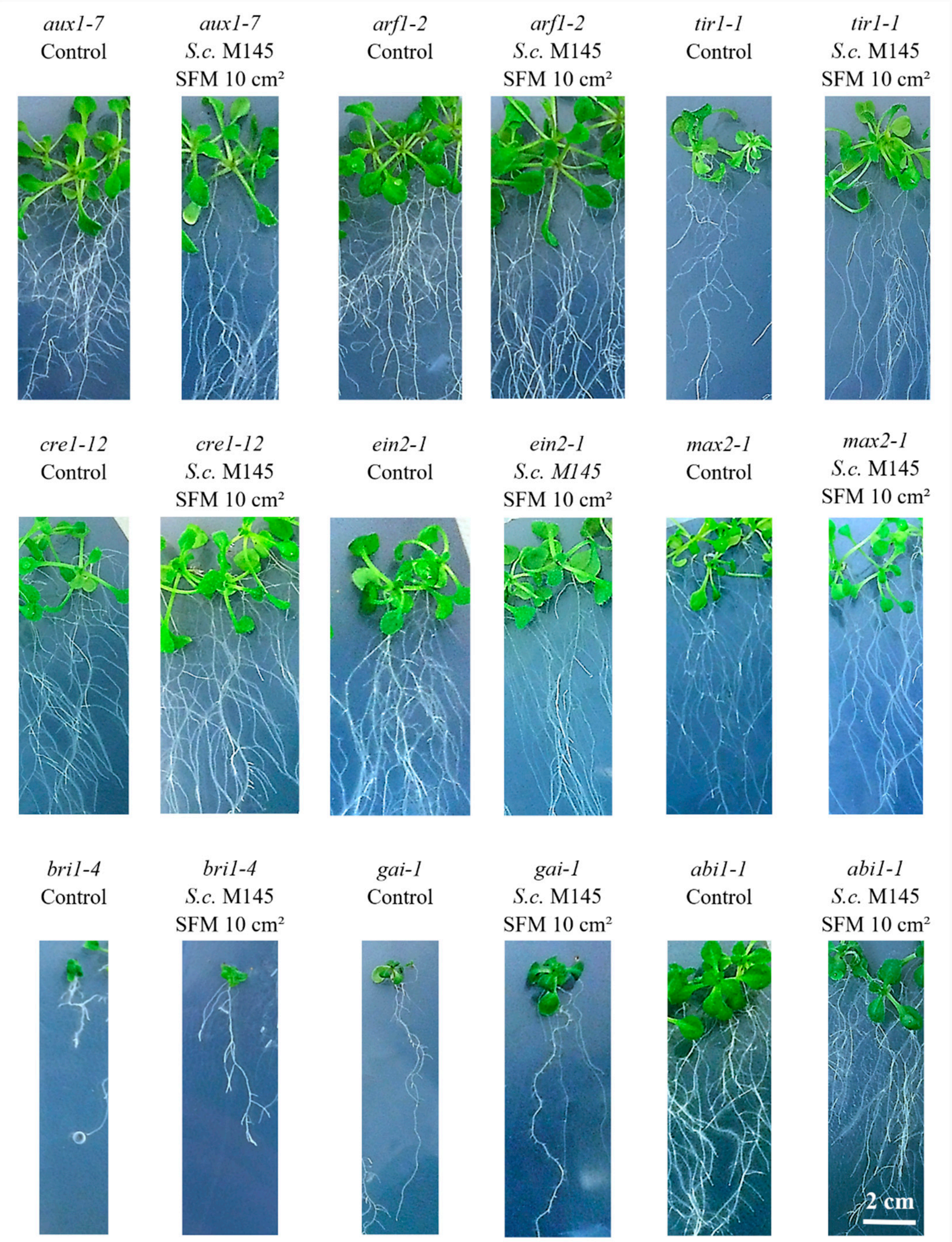

Figure S2. Growth of *Arabidopsis thaliana* hormone mutants in response to *Streptomyces coelicolor* M145 volatiles**.** Seven-day-old seedlings of *Arabidopsis thaliana* hormone mutants were treated ± volatiles from 10 cm² of *Streptomyces coelicolor* M145 culture on SFM for additionally six days.

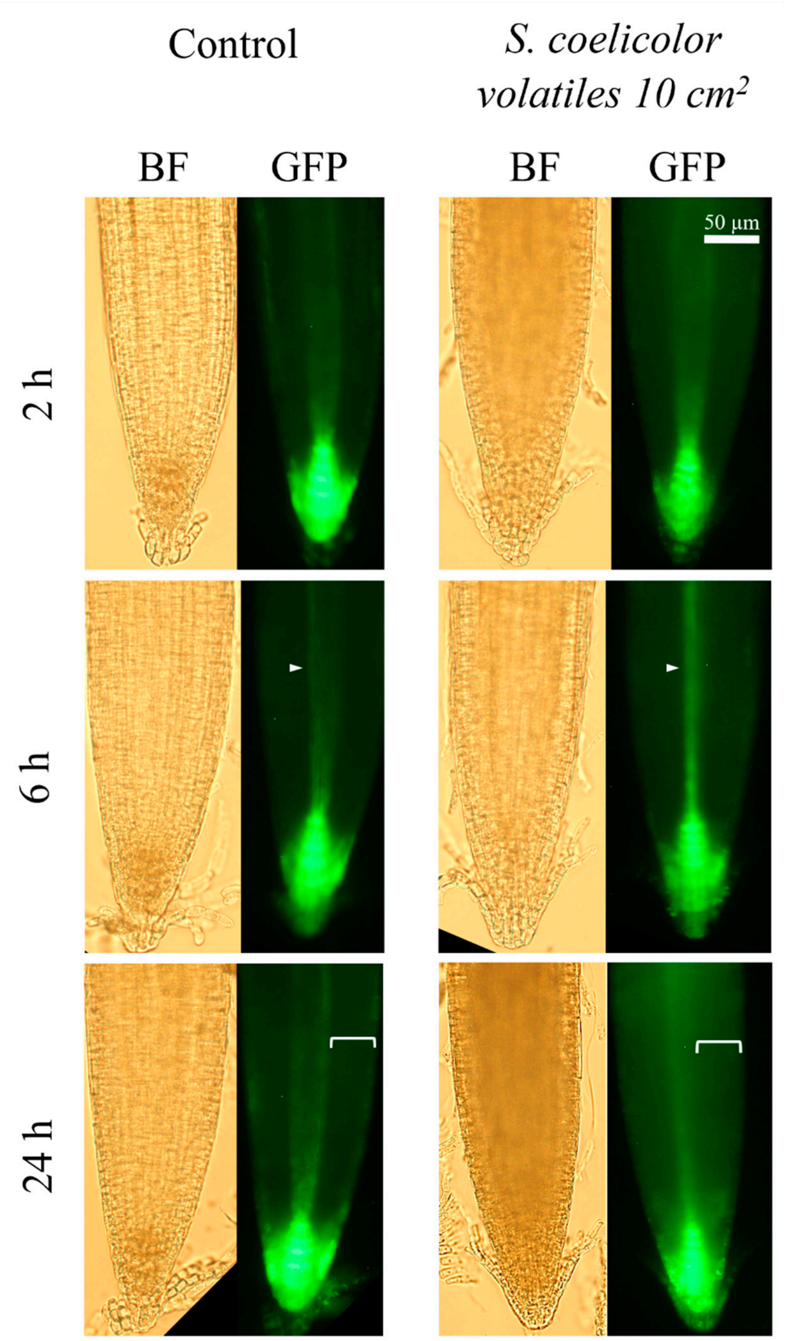

Figure S3. *Arabidopsis thaliana* DR5::GFP response to *Streptomyces coelicolor* volatiles. Representative figures of primary root tips of the DR5::GFP line treated over different times ± *S. coelicolor* volatiles are shown. Seven-day-old DR5-GFP seedlings were exposed for 2, 6 and 24 h to volatiles from S. coelicolor M145 grown on 10 cm^2 SFM medium or to control plates. Roots were analyzed by bright field and fluorescence microscopy using a GFP-filter (excitation at 457 - 487 nm, emission 502 - 538 nm) coupled to a Nikon-Optiphot-2 microscope (Nikon Corporation, Tokyo, Japan). DR5::GFP expression visualizes a redistribution of the auxin response upon exposure to S. coelicolor volatiles. DR5::GFP shows little difference in fluorescence distribution after 2 h of S. coelicolor volatiles as compared to control. However, after 6 h treatment, an increase in fluorescence could be observed in the vascular tissue relative to the apical meristem (cf. arrowheads) and after 24 h a general tissue-wide diffuse fluorescence was specifically observed in the volatile-treated root (brackets).

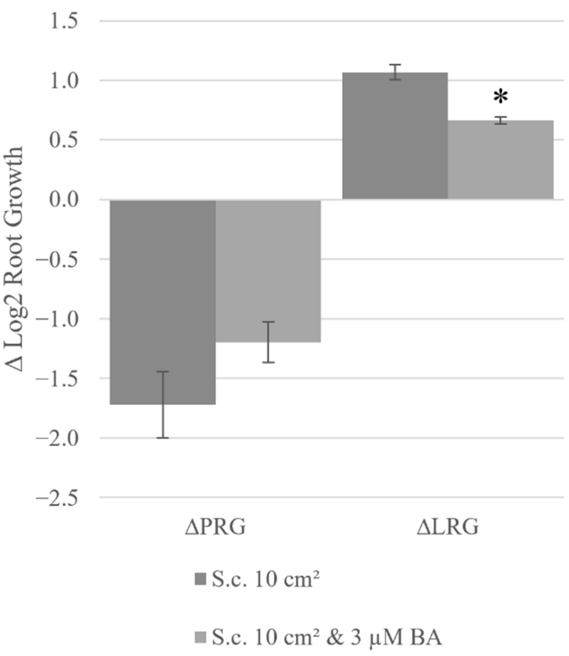

Figure S4. Root growth response of *Arabidopsis thaliana* Col-0 seedlings exposed to *Streptomyces coelicolor* volatiles with cytokinin. Seven-day-old *Arabidopsis thaliana* Col-0 seedlings root growth after two days ± exposure to volatiles from 10 cm² sized plates containing *Streptomyces coelicolor* (*S.c.*) M145 compared to root growth on plates containing 3 µM of the cytokinin benzyladenine (BA). Growth rate was calculated as root length extension for control and volatile-exposed seedlings. Log2 ratio of control to volatile treated Primary Root Growth (PRG) and Lateral Root Growth (LRG) are given as means ± standard error and asterisks indicate significant differences to the control treatment (0 µM BA + *S.c.* volatiles) according to Student's *t*-test with false discovery rate (FDR) correction for $q = 0.05$.

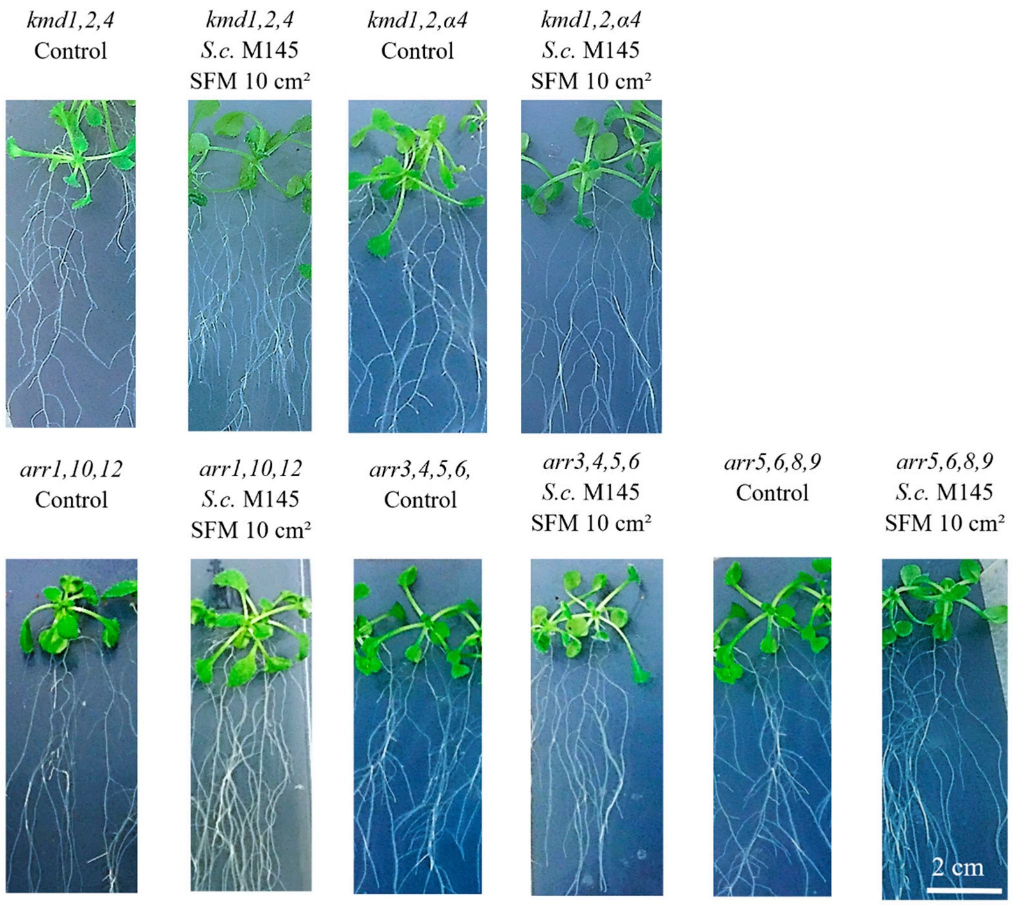

Figure S5. Growth of *Arabidopsis thaliana* mutants for *KMD*-associated genes in response to *Streptomyces coelicolor* volatiles. The images show representative 7-day-old seedlings of *A. thaliana KMD* and associated cytokinin signaling mutants that have been treated ± volatiles from 10 cm² of *S. coelicolor* M145 for an additional six days.

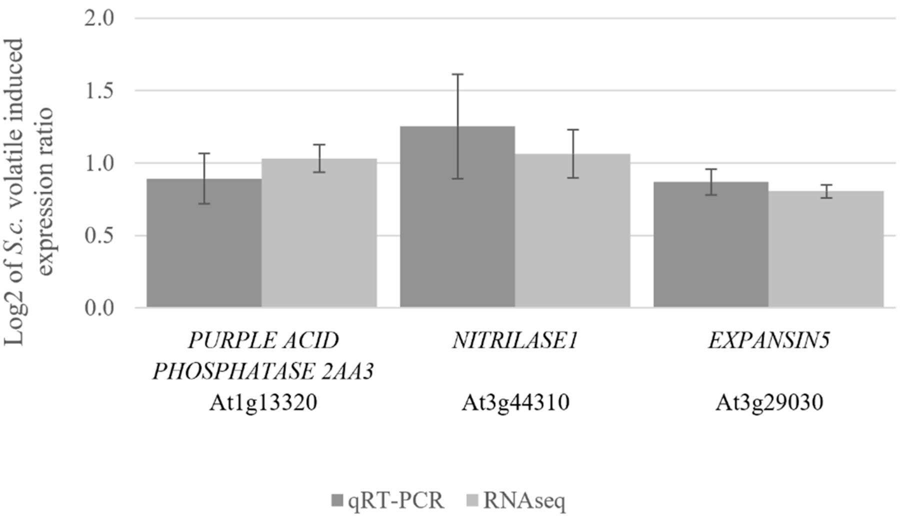

Figure S6. qRT-PCR verification of RNA-Seq expression. Comparison of transcript ratios between 7-day-old Col-0 seedlings that have been exposed to volatiles from a 10 cm^2 culture of *Streptomyces coelicolor* M145 for 2 h and control seedlings. Comparison of expression values was made by qRT-PCR and RNA-Seq. Each gene signal was normalized to the *UBIQUITIN PROTEIN LIGASE7* (At3g53090) internal control gene in the qRT-PCR analysis. Bars represent standard errors (n=3).

Table S1. Growth data for Col-0 response to gas-phase connection to different amounts of each *Streptomyces* strain

**Experimental conditions**	**LRG (cm) day^{-1} LR^{-1}**	**PRG (cm) day^{-1} PR^{-1}**	**log2 PRG/LRG**	**LRE (count) day^{-1} PR^{-1}**
Control	0.19 ± 0.004	0.97 ± 0.01	2.35 ± 0.03	3.91 ± 0.11
2 cm² *S.c.* M145	0.22 ± 0.02	**0.77 ± 0.05**	**1.81 ± 0.03**	3.66 ± 0.04
5 cm² *S.c.* M145	**0.25 ± 0.01**	**0.64 ± 0.04**	**1.35 ± 0.07**	3.79 ± 0.11
10 cm² *S.c.* M145	**0.40 ± 0.01**	**0.34 ± 0.01**	**-0.25 ± 0.03**	3.74 ± 0.13
2 cm² *S.c. bldA*	0.26 ± 0.03	0.81 ± 0.08	**1.62 ± 0.10**	3.79 ± 0.06
5 cm² *S.c. bldA*	**0.42 ± 0.03**	0.77 ± 0.06	**0.86 ± 0.03**	4.19 ± 0.23
10 cm² *S.c. bldA*	**0.31 ± 0.02**	**0.18 ± 0.02**	**-0.78 ± 0.08**	3.89 ± 0.12
2 cm² *S.c. bldM*	0.23 ± 0.03	1.00 ± 0.04	2.13 ± 0.22	3.11 ± 0.39
5 cm² *S.c. bldM*	0.38 ± 0.03	**0.44 ± 0.01**	**0.56 ± 0.11**	3.56 ± 0.24
10 cm² *S.c. bldM*	**0.46 ± 0.02**	**0.51 ± 0.02**	**0.13 ± 0.07**	4.23 ± 0.08
2 cm² *S.c. geoA mibAB*	0.21 ± 0.02	1.06 ± 0.06	2.33 ± 0.22	4.31 ± 0.37
5 cm² *S.c. geoA mibAB*	**0.38 ± 0.02**	**0.73 ± 0.02**	**0.94 ± 0.06**	4.28 ± 0.39
10 cm² *S.c. geoA mibAB*	**0.29 ± 0.01**	**0.22 ± 0.02**	**-0.40 ± 0.09**	3.76 ± 0.07
2 cm² *S.v.*	0.21 ± 0.01	**0.88 ± 0.02**	**2.05 ± 0.08**	3.50 ± 0.06
5 cm² *S.v.*	0.21 ± 0.01	**0.81 ± 0.01**	**1.92 ± 0.08**	3.43 ± 0.07
10 cm² *S.v.*	0.18 ± 0.01	**0.85 ± 0.01**	2.22 ± 0.05	3.37 ± 0.03

Seven-day-old *Arabidopsis thaliana* Col-0 seedlings were exposed for two days to volatiles emanating from differently sized plates containing *S. coelicolor* (*S.c.*) genotypes or *S. venezuelae* (*S.v.*), grown on SFM and MYM media, respectively. Growth rate was calculated as root length extension for control and volatile-exposed seedlings. Lateral Root Growth (LRG) per Lateral Root (LR), Primary Root Growth (PRG) per Primary Root (PR), the log2 ratio between PRG and LRG (PRG/LRG) and Lateral Root Emergence (LRE) per primary root are given as mean ± standard error. Bold font indicates significant differences to the control treatment according to Student's t-test with false discovery rate (FDR) correction for $q = 0.05$.

Table S2. Transcriptional comparison of *Arabidopsis thaliana* Col-0 treated with *Streptomyces coelicolor* volatiles to exogenous hormone application

**Publication**<br>Treatment	**Dotson et al. 2026**<br>10 cm² of *S.c.* volatiles for 2 hr	**Omelyanchuk et al. 2017**<br>1 µM of IAA for 6 hr	**Bhargava et al. 2013**<br>5 µM of BA for 2 hr
CYTOKININ BINS			
GO-0080037 negative regulation of cytokinin-activated signaling pathway	0.40	-0.37	-0.14
GO-0080036 regulation of cytokinin-activated signaling pathway	0.21	-0.16	0.04
GO-0009691 cytokinin biosynthetic process	0.15	-0.05	-0.01
GO-0009824 AMP dimethylallyl transferase activity	0.10	0.29	0.03
GO-0009690 cytokinin metabolic process	0.07	0.35	0.43
GO-0009736 cytokinin-activated signaling pathway	0.07	0.16	0.59
GO-0009823 cytokinin catabolic process	0.06	1.03	1.38
GO-0009735 response to cytokinin	0.04	0.01	0.50
GO-0071368 cellular response to cytokinin stimulus	0.03	0.19	0.65
GO-0009884 cytokinin receptor activity	0.02	N.A.	0.47
GO-0019139 cytokinin dehydrogenase activity	0.00	1.04	1.17
AUXIN BINS			
GO-0010929 positive regulation of auxin mediated signaling pathway	0.25	0.44	-0.28
GO-0010600 regulation of auxin biosynthetic process	0.18	-0.94	-0.08
GO-0090354 regulation of auxin metabolic process	0.15	-0.76	-0.09
GO-0010178 IAA-amino acid conjugate hydrolase activity	0.10	-0.48	0.00
GO-0009851 auxin biosynthetic process	0.08	-0.31	0.02
GO-0009850 auxin metabolic process	0.07	-0.25	-0.01
GO-0009733 response to auxin	0.07	0.51	0.00
GO-0080024 indole butyric acid metabolic process	0.07	0.31	-0.20
GO-0060774 auxin mediated signaling pathway involved in phyllotactic patterning	0.07	2.53	0.00
GO-0010928 regulation of auxin mediated signaling pathway	0.06	0.55	0.04
GO-0009734 auxin-activated signaling pathway	0.06	0.63	-0.09
GO-0071365 cellular response to auxin stimulus	0.05	0.61	-0.06
GO-0010011 auxin binding	0.04	-0.01	-0.17
GO-0010315 auxin efflux	0.02	0.53	0.11
GO-0009852 auxin catabolic process	0.02	-1.83	0.10
GO-2000012 regulation of auxin polar transport	0.02	-0.27	-0.07
GO-0010329 auxin efflux transmembrane transporter activity	0.02	0.70	0.10
GO-0060918 auxin transport	0.01	0.26	-0.05
GO-0080161 auxin transmembrane transporter activity	0.01	0.83	0.06
GO-0010252 auxin homeostasis	0.00	0.80	-0.08
GO-0009926 auxin polar transport	0.00	0.31	-0.08
GO-0090355 positive regulation of auxin metabolic process	0.00	0.13	-0.23
GO-0010601 positive regulation of auxin biosynthetic process	-0.01	0.09	-0.27
GO-0010541 acropetal auxin transport	-0.03	0.60	-0.20
GO-0010328 auxin influx transmembrane transporter activity	-0.04	0.75	-0.03
GO-0010540 basipetal auxin transport	-0.05	0.32	-0.15
GO-0060919 auxin influx	-0.06	0.75	0.04

Analyses of the volatile responsivity of cytokinin- and auxin-associated GO bins by comparison of average log2 responses to respective treatments. Cells are colored by strength of positive (red) or negative (blue) differential average log2 responses. GO terms are as annotated by AmiGO [36].

Table S3. Genetic stocks

Name	Relevant Gene Mutations	Species, Ecotype or Parental Isolates	References
Col-0	N.A. (Wild-type)	*Arabidopsis thaliana* Col-0	N.A.
arf1-2	At1g59750	*Arabidopsis thaliana* Col-0	[71]
arr1-3,10-1,12-1	At3g16857, At4g31920, At2g25180	*Arabidopsis thaliana* Col-0	[72]
arr3-1,4-1,5-1,6-1	At1g59940, At1g10470, At3g48100, At5g62920	*Arabidopsis thaliana* Col-0	[73]
arr5-1,6-1,8-1,9-1	At3g48100, At5g62920, At2g41310, At3g57040	*Arabidopsis thaliana* Col-0	[73]
aux1-7	At2g38120	*Arabidopsis thaliana* Col-0	[74]
cre1-12	At2g01830	*Arabidopsis thaliana* Col-0	[75]
DR5::GFP	pDR5::GFP (construct)	*Arabidopsis thaliana* Col-0	[76]
ein2-1	At5g03280	*Arabidopsis thaliana* Col-0	[77]
etr1-1	At1g66340	*Arabidopsis thaliana* Col-0	[78]
kmd1-1,2-1,4-1	At1g80440, At1g15670, At3g59940	*Arabidopsis thaliana* Col-0	[23]
kmd1-1,2-1,α4	At1g80440, At1g15670, (At2g44130), At3g59940	*Arabidopsis thaliana* Col-0	[23]
max2-1	At2g42620	*Arabidopsis thaliana* Col-0	[79]
tir1-1	At3g62980	*Arabidopsis thaliana* Col-0	[80]
abi1-1	At4g26080	*Arabidopsis thaliana* Ler-0	[81]
gai-1	At1g14920	*Arabidopsis thaliana* Ler-0	[82]
bri1-4	At4g39400	*Arabidopsis thaliana* Ws-2	[83]
M145-(*S.c.*)	Prototrophic, $SCP1^-$ $SCP2^-$	*Streptomyces coelicolor* A3(2)-(M145)	[54]
J2192-(*geoA mibAB*)	Δ*geoA* Δ*mibAB::apr*	*Streptomyces coelicolor* A3(2)-(M145)	[84]
J1681-(M600)	Prototrophic, $SCP1^-$ $SCP2^-$	*Streptomyces coelicolor* A3(2)-(M600)	[54]
J1681-(*bldA)*	Δ*bldA::apr*	*Streptomyces coelicolor* A3(2)-(M600)	[85]
J3445-(*bldM*)	Δ*bldM::apr*	*Streptomyces coelicolor* A3(2)-(M600)	[54]
NRRL B-65442-(*S.v.*)	Wild-type	*Streptomyces venezuelae* (NRRL B-65442)	[86]

Genotypes of *Arabidopsis thaliana* seed lines and *Streptomyces* isolates used in this publication.

Table S4. Primer sequences

Target Gene	Forward	Reverse	Product Size
UPL7	TGCTACAATACTCTTAAGCTTCCAACG	GTGCATAACAAGATGAATACCTGGTT	136
PP2AA3	ACCAGCTGAAAGTCGCTTAGC	GCTATGGCGGAAGAGTTGGG	213
EXP5	CCGCATGCTCCACCCATAG	CGCTTCTCGTGGTTCATCTCC	160
NIT1	TCGTCACAGCTGATATTGATATAGC	TCCTCGGGTGCTCATTTACGG	142

Gene targets, forward and reverse primer sets and product sizes for qRT-PCR verification.
